# DAPCy: a Python package for the discriminant analysis of principal components method for population genetic analyses

**DOI:** 10.1093/bioadv/vbaf143

**Published:** 2025-06-18

**Authors:** Alejandro Correa Rojo, Pieter Moris, Hanne Meuwissen, Pieter Monsieurs, Dirk Valkenborg

**Affiliations:** Data Science Institute, Interuniversity Institute for Biostatistics and Statistical Bioinformatics (I-BioStat), Hasselt University, Diepenbeek 3500, Belgium; Flemish Institute for Technological Research (VITO), Mol 2400, Belgium; Unit of Malariology, Department of Biomedical Sciences, Institute of Tropical Medicine, Antwerp 2000, Belgium; Data Science Institute, Interuniversity Institute for Biostatistics and Statistical Bioinformatics (I-BioStat), Hasselt University, Diepenbeek 3500, Belgium; Unit of Malariology, Department of Biomedical Sciences, Institute of Tropical Medicine, Antwerp 2000, Belgium; Flemish Institute for Technological Research (VITO), Mol 2400, Belgium

## Abstract

**Summary:**

The Discriminant Analysis of Principal Components method is a pivotal tool in population genetics, combining principal component analysis and linear discriminant analysis to assess the genetic structure of populations using genetic markers, focusing on the description of variation between genetic clusters. Despite its utility, the original R implementation in the adegenet package faces computational challenges with large genomic datasets. To address these limitations, we introduce DAPCy, a Python package leveraging the scikit-learn library to enhance the method’s scalability and efficiency. DAPCy supports large datasets by utilizing compressed sparse matrices and truncated singular value decomposition for dimensionality reduction, coupled with training-test cross-validation for robust model evaluation. It also includes modules for *de novo* genetic clustering and extensive visualization and reporting capabilities. Compared to the original R implementation, DAPCy can process genomic datasets with thousands of samples and features in less computational time and with reduced memory usage. To show DAPCy’s computational capabilities, we benchmarked it with the R implementation using the *Plasmodium falciparum* dataset from MalariaGEN and the 1000 Genomes Project.

**Availability and implementation:**

DAPCy can be installed as a Python package through pip. Source code is available on https://gitlab.com/uhasselt-bioinfo/dapcy. Documentation and a tutorial can be found on https://uhasselt-bioinfo.gitlab.io/dapcy/.

## 1 Introduction

Over a decade ago, Jombart *et al.* (2010) introduced Discriminant Analysis of Principal Components (DAPC), a pivotal method for analyzing genetically structured populations (Jombart *et al.* 2010). DAPC combines principal component analysis (PCA) and linear discriminant analysis (DA) to reduce dimensionality and identify clusters of genetically related individuals. Initially implemented in R with the package adegenet (https://github.com/thibautjombart/adegenet) (Jombart 2008, Jombart and Ahmed 2011), DAPC has garnered thousands of citations, attesting to its enduring utility in population genetic research. In DAPC, genotype data is transformed using PCA to yield uncorrelated principal components (PCs) that are then used in DA to maximize variation between pre-defined groups while minimizing within-group variation. In addition, as a preliminary step for the DAPC method and included in the adegenet package, K-means clustering is frequently employed to infer the effective number of genetic clusters when prior group information is unavailable. More recently, derivatives of the DAPC method that incorporate Kernel techniques and Local Fisher Discriminant Analysis have been proposed to capture more complex patterns of population structure (Qin *et al.* 2021, 2022). Yet, the classical DAPC method remains popular, supported by established guidelines for its proper implementation and the accurate analysis of population structure inferred from DAPC (Miller *et al.* 2020, Cullingham *et al.* 2023, Thia 2023).

Despite the success of DAPC, the original R implementation (as bundled in the adegenet package) requires substantial computational resources when analyzing datasets with thousands of genetic variants such as marker alleles or single nucleotide polymorphisms (SNPs) across numerous sample sizes. With the advent of next-generation sequencing technologies such as whole-genome sequencing, more available genetic datasets include large to extreme sample and SNP sizes for analyses. For most applications, DAPC is still effective for small- to medium-sized datasets with fewer than thousands of alleles or feature variables. However, for larger genomic datasets, the application of DAPC can be slow or computationally prohibitive due to the inherent limitations of R’s memory management and the lack of optimized, low-level implementations for certain operations (Visser *et al.* 2015). Although the R ecosystem offers several packages for storage and PCA scalable analysis for large genomic datasets, such as included in Bioconductor (https://bioconductor.org/), or RSpectra (https://github.com/yixuan/RSpectra), these alternatives have not yet been integrated into adegenet’s DAPC workflow. Moreover, for estimating the PCs, the R implementation relies on eigendecomposition for PCA (Paradis 2020), which scales quadratically in terms of time complexity for datasets where the number of features is larger than the sample size, as is often the case for genetic datasets. Hence, this increases runtime for large datasets (Agrawal *et al.* 2020).

To address these computational limitations, we introduce DAPCy, a re-implementation of the DAPC method in Python using the scikit-learn (https://scikit-learn.org) machine learning (ML) library. DAPCy enhances scalability in population genetic analyses by empowering sparse matrix algebra, enabling deployment in resource-constrained environments. Additionally, this package provides greater flexibility in model training by allowing a choice of cross-validation schemes for hyperparameter tuning and model assessment. Finally, DAPCy extends the utility of the DAPC method by offering a portable machine learning classifier in addition to its exploratory capabilities.

## 2 Overview of DAPCy

**Figure 1.**
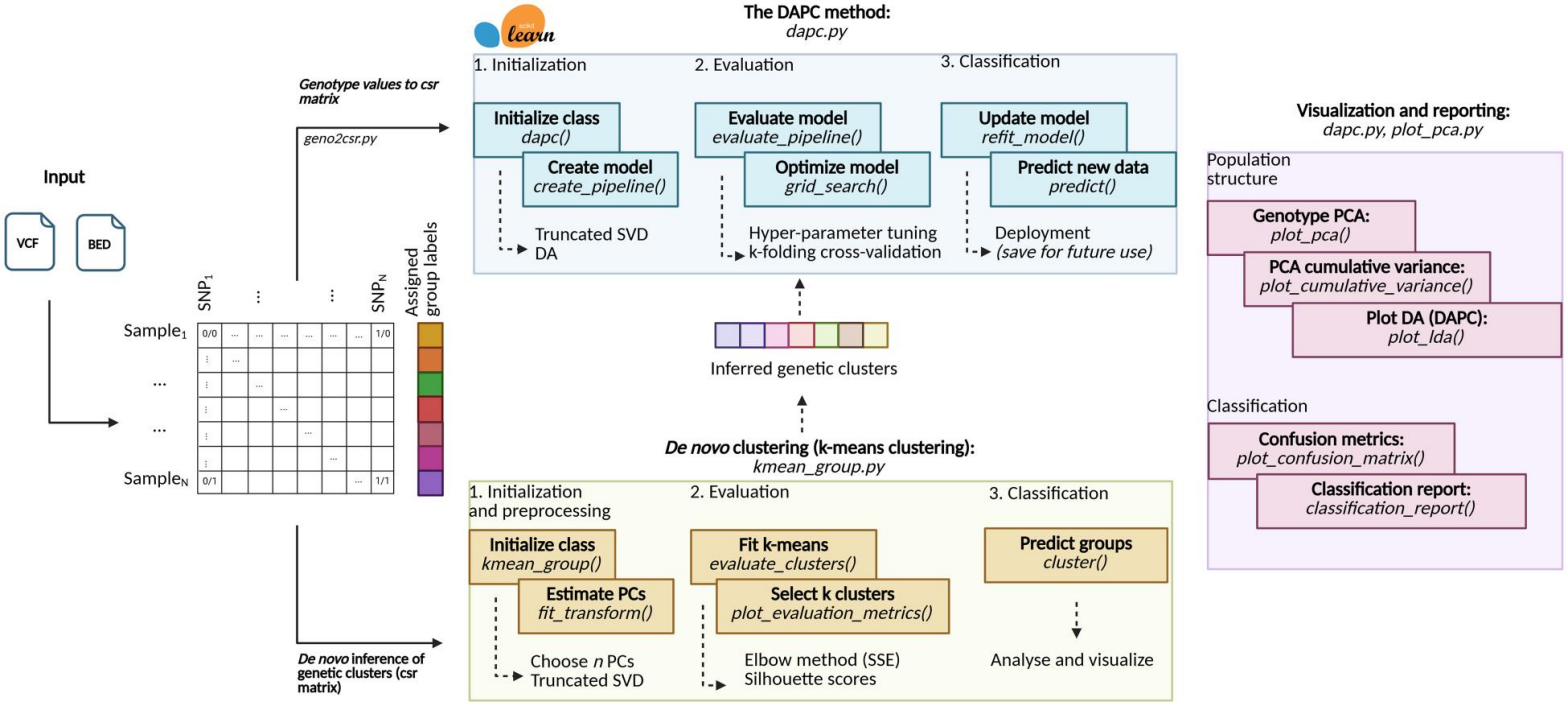
Overview of DAPCy. DAPCy is a Python package that uses scikit-learn to apply the DAPC method to a genotype matrix. It takes a VCF or BED file as input and transforms it into a csr matrix. The DAPC method is encoded as an automated ML pipeline that provides all functions for training/estimation, model performance assessment through training-test cross-validation, grid-search cross-validation for hyper-parameter tuning, and deployment of the final model. DAPCy includes a module for *de novo* analysis of genetic clusters using the K-means clustering algorithm, allowing users to infer genetic groups from the PCs if population or location data is unavailable. Finally, DAPCy includes functions for visualization and reporting, including scatter plots, accuracy test reports, and confusion matrices. Image created with BioRender.com.

DAPCy is a custom ML workflow that uses scikit-learn’s API, designed for fast and robust analysis of large genomic datasets. As key features, DAPCy first reads in genomic data (stored in VCF or BED files) and extracts the genotype values of the samples as a compressed sparse (csr) matrix to reduce memory consumption. Next, DAPCy estimates the PCs using truncated SVD and applies DA to the approximated components to speed up computation. For model evaluation and robustness, DAPCy relies on a training-test cross-validation scheme to assess the performance of the DA and grid-search cross-validation for hyper-parameter tuning. In addition to classification tasks, DAPCy includes functions for reporting, visualization, and K-means clustering for *de novo* designation of populations. An overview of the DAPCy workflow is illustrated in Fig. 1.

## 3 Implementation

### 3.1 Data preparation and transformation

DAPCy includes a built-in function (“geno2csr.py”) that extracts the genotype values from VCF or BED files using the sgkit (https://sgkit-dev.github.io/sgkit) library. For VCF files, DAPCy processes the input VCF file chunk-wise, converting each chunk into compressed zarr files and extracting the values. For BED files, DAPCy extracts the genotype matrix directly from the input. Genotype values are then transformed into a csr matrix using the SciPy (https://scipy.org/) library for efficient arithmetic operations and reduced memory storage.

### 3.2 The DAPC method

DAPCy provides a class for the DAPC method (“dapc.py”) with built-in functions for training, cross-validation, visualization, and reporting. We implement an automated pipeline for classifying the csr matrix into the assigned population groups (either known a priori or estimated using *de novo* K-means clustering). Contrary to the R implementation, which applies eigendecomposition for estimating the PCs, we introduced a truncated SVD to efficiently handle larger matrices (Falini 2022). A truncated SVD performs PCA on a sparse matrix by only retaining the most significant eigenvalues and eigenvectors (Supplementary data, available as supplementary data at *Bioinformatics Advances* online). Using this approach, DAPCy allows the analysis of large genotype matrices by making the DAPC method more computationally efficient, speeding up computations, and reducing memory consumption.

### 3.3 Cross-validation and model evaluation

In DAPCy, we implement training-test partitioning schemes based on cross-validation for accuracy testing and model evaluation, including $k_{\text{CV}}$-fold cross-validation, stratified $k_{\text{CV}}$-fold cross-validation, and leave-one-out cross-validation (LOOCV). To avoid confusion with the number of groups or effective populations (denoted as *k*), we denote the number of cross-validation folds by $k_{\text{CV}}$. This design overcomes limitations in adegenet, which rely on bootstrapping and may incur high variance and heavy computational demands for large datasets (Kim 2009). Standard $k_{\text{CV}}$-fold partitions the data into $k_{\text{CV}}$ folds, trains on $k_{\text{CV}}-1$ folds, and tests on the remaining fold, whereas stratified $k_{\text{CV}}$-fold preserves proportional representation in each fold for imbalanced datasets. In contrast, LOOCV tests each sample individually, maximizing training data but increasing both computational cost and the variance of performance estimates. As such, LOOCV is typically more suitable for smaller datasets, while standard or stratified $k_{\text{CV}}$-fold approaches provide more stable performance and reduced computational burden for larger datasets (Thia 2023). Finally, DAPCy employs an automated grid-search cross-validation to select the optimal number of PCs with the highest accuracy metric (percentage correct between predicted and actual class labels) without overfitting.

### 3.4 K-means clustering for *de novo* inference of genetic groups

DAPCy provides a K-means clustering pipeline with built-in functions for automated model optimization (“kmean_group.py”), enabling users to infer the expected number of population groups prior to DAPC ($k_{\text{infer}}$). By default, DAPCy uses the sum of squared errors (SSE) or Silhouette scores to evaluate different cluster solutions (Supplementary data, available as supplementary data at *Bioinformatics Advances* online), whereas the R adegenet package employs Bayesian Information Criterion (BIC) values. However, because K-means is a model-driven method, the “optimal” number of clusters can depend heavily on user-defined parameters, which can lead to misinterpretation in population structure predictions (Miller *et al.* 2020, Cullingham *et al.* 2023, Thia 2023). To guide users through these considerations, such as inferring population groups and selecting the optimal number of PCs, DAPCy provides a tutorial to avoid biased results, following the guidelines provided by Thia (2023).

### 3.5 Visualization, reporting, and deployment

DAPCy provides several functions to plot the results from the DAPC method and generates classification reports to assess the performance of the model as an ML classifier. For instance, DAPCy generates scatterplots of the results and reports accuracy scores for each cluster, as well as the overall mean accuracy of the classifier. Additionally, with DAPCy, users can create, train, and export the classifier as a pickle file (.pkl). This allows models to be deployed across different environments and workstations without the need for re-training. We provided documentation and tutorials at https://uhasselt-bioinfo.gitlab.io/dapcy/.

### 3.6 Benchmarking

**Figure 2.**
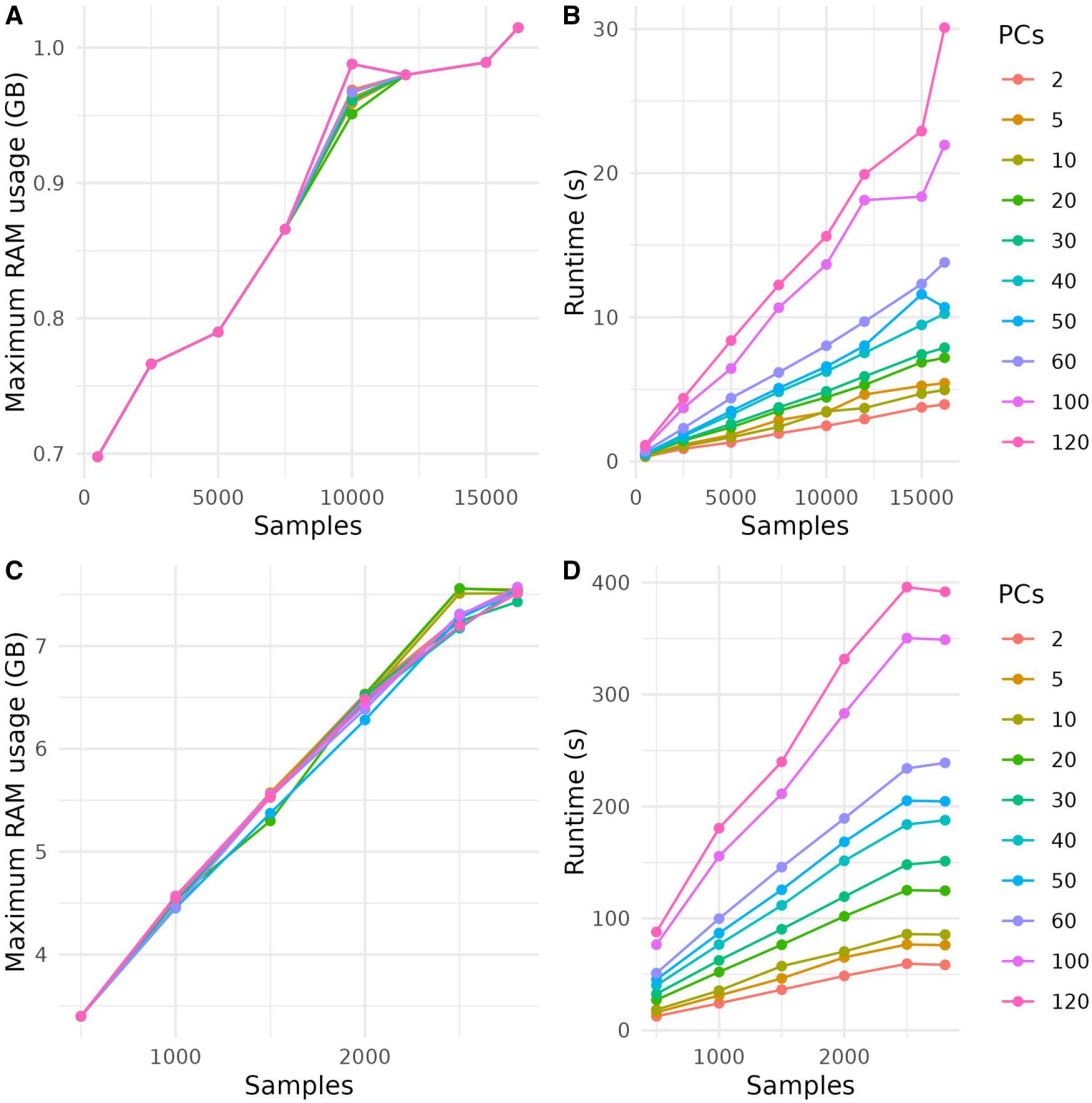
Performance of DAPCy on the Pf7 ($N_{\text{SNPs}}=6,385$) and 1KG ($N_{\text{SNPs}}=359,158$) datasets as a function of sample size at different PCs. (A) Memory usage (GB) for the Pf7 dataset. (B) Runtime (s) for Pf7 dataset. (C) Memory usage (GB) for the 1KG dataset. (D) Runtime (s) for the 1KG dataset.

To evaluate DAPCy for large-scale analyses and assess its computational performance, we used two genomic variant datasets, *Plasmodium falciparum* from MalariaGEN (Pf7; *N* = 16 203) (MalariaGEN 2023) and the 1000 Genomes Project (1KG; *N* = 2805) (The 1000 Genomes Project Consortium 2015). VCF files were converted to BED format using PLINK (https://www.cog-genomics.org/plink), and SNPs with a minor allele frequency below 10% and linkage disequilibrium above $r^{2}=0.3$ were filtered out, yielding 6385 uncorrelated SNPs for Pf7 and 359 130 SNPs for 1KG. Additional details on these benchmarking and classification procedures are provided in the Supplementary data, available as supplementary data at *Bioinformatics Advances* online. We first assessed DAPCy’s runtime and memory usage by classifying samples (using country of origin for Pf7 and genetic population labels for 1KG) with up to 120 PCs, thereby stress-testing its performance with a high number of PCs as input parameters. As shown in Fig. 2 and the Supplementary data, available as supplementary data at *Bioinformatics Advances* online, DAPCy efficiently processed both datasets without exceeding 10 GB of RAM.

**Figure 3.**
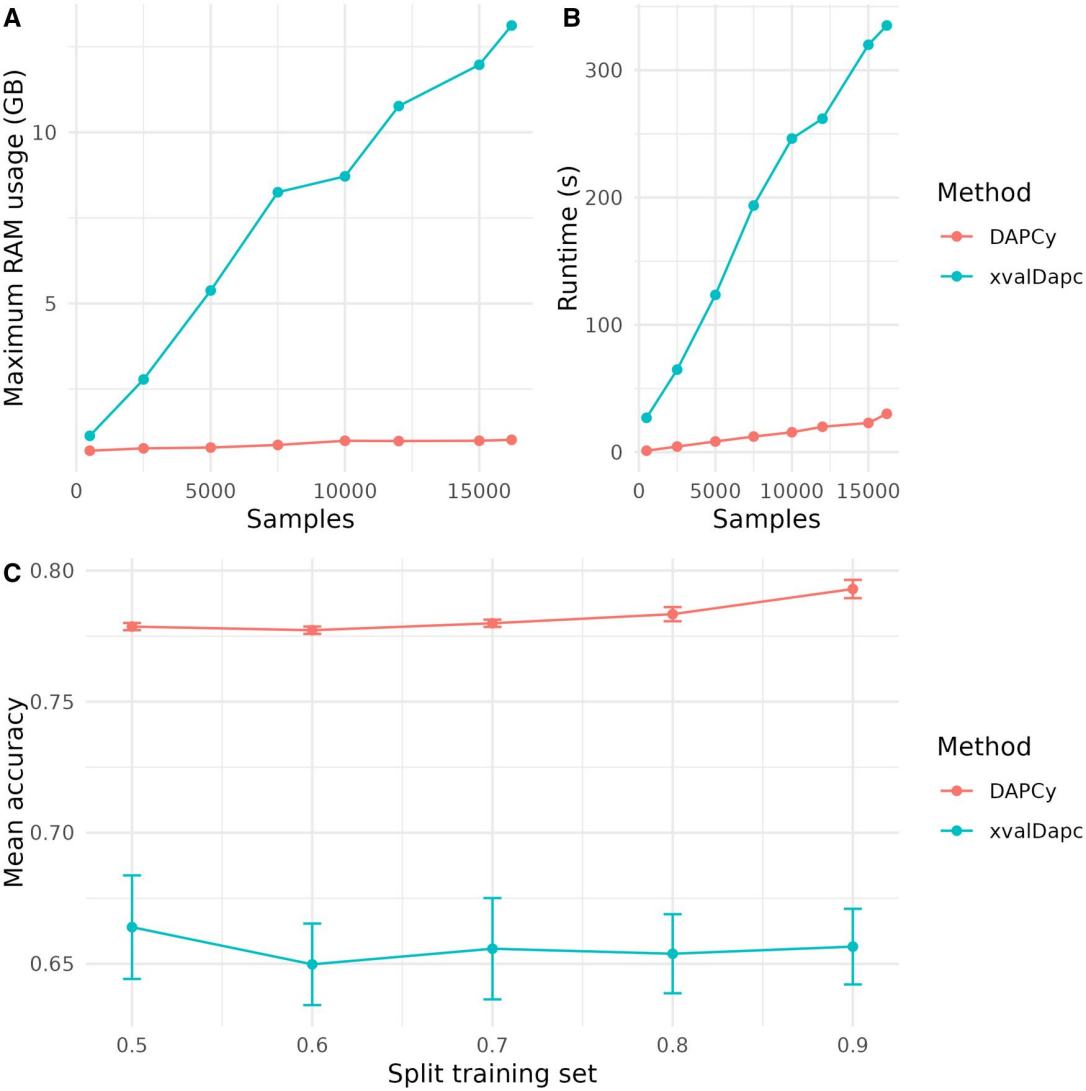
Benchmarking between DAPCy and the R adegenet implementation of the DAPC method on the Pf7 dataset ($N_{\text{SNPs}}=6,385$). (A) Memory usage (GB). (B) Runtime (s). (C) Mean accuracy estimates at different training splits. Cross-validation was performed with $k_{\text{CV}}$ = 10.

Next, we benchmarked DAPCy’s performance and cross-validation strategy ($k_{\text{CV}}$-fold) against the R implementation of the DAPC method in adegenet using the “xvalDapc()” function with bootstrapping. For the Pf7 dataset, DAPCy was 14.26 times faster and more memory efficient than adegenet (Fig. 3A and B); for the 1KG dataset, “xvalDapc()” could not be run at all, as it required over 45 GB of RAM. For Pf7, we also evaluated mean accuracy across training set sizes ranging from 50% to 90% of the full dataset. DAPCy consistently provided higher and more robust estimates compared to the bootstrapping-based approach of “xvalDapc(),” which produced lower accuracy scores with high variance (Fig. 3C). As noted by Thia (2023), the “xvalDapc()” function can struggle to determine the optimal number of PCs, a limitation that aligns with our observations of lower accuracy and high variance (Kim 2009). By employing $k_{\text{CV}}$-fold cross-validation, DAPCy delivers a more reliable performance assessment while avoiding the variance and biases often introduced by bootstrapping in the R implementation.

Finally, following the guidelines from Thia (2023) for population structure analyses using the DAPC method, we conducted classification analyses on both datasets by first describing population structure using the population labels included in the Pf7 (country of origin; k=33) and 1KG (genetic population groups; k=5) datasets. In addition, for the Pf7 dataset, we performed a *de novo* inference of population groups via K-means clustering, applying the k−1 criterion for selecting the optimal number of principal components prior to the DAPC step (Patterson *et al.* 2006). Using the sample labels with grid-search $k_{\text{CV}}$-fold cross-validation, DAPCy achieved classification accuracies of 71.86% for the Pf7 dataset (country of origin labels) and 97.50% for the 1KG dataset (genetic population labels). Moreover, the *de novo* K-means clustering on Pf7 inferred four effective populations ($k_{\text{infer}}=4$), which increased the classification accuracy to 95.76% when using the first three PCs derived from K-means clustering, which was the optimal number based on the k−1 criterion. In both the 1KG dataset and the *de novo* Pf7 model, the first two discriminant components displayed clinal distributions consistent with previous studies (The 1000 Genomes Project Consortium 2012, MalariaGEN 2023), as detailed in the Supplementary data, available as supplementary data at *Bioinformatics Advances* online and the Pf7 tutorial.

## 4 Conclusions

We present DAPCy, a re-implementation of the DAPC method from the R package adegenet, used in population genetic research for identifying and describing genetic clusters. DAPCy, written in Python and using the scikit-learn framework, supports VCF and BED files and includes an automated ML pipeline for model training, evaluation, visualization, and classification reports. Python’s efficiency and scikit-learn’s portable classifiers make DAPCy particularly well-suited for large genomic datasets.

To optimize for the sparse nature of genomic data, DAPCy employs the truncated SVD as PCA, significantly reducing computational requirements. It also replaces the bootstrapped cross-validation of the original R implementation with $k_{\text{CV}}$-fold schemes, including stratified $k_{\text{CV}}$-fold and leave-one-out cross-validation, providing more reliable performance metrics and enabling more informed parameter selection. Together, these improvements allow DAPCy to process much larger genomic datasets, turning analyses that were once prohibitive into practical, resource-efficient workflows.

## Supplementary Material

vbaf143_Supplementary_Data

## Data Availability

For evaluation and benchmarking of DAPCy, we used the public datasets of *Plasmodium falciparum* version 7 from MalariaGEN (ftp://ngs.sanger.ac.uk/production/malaria/Resource/34/Pf7_vcf/) and the 1000 Genomes Project (https://ftp.1000genomes.ebi.ac.uk/vol1/ftp/release/20130502/).
