# Supplementary material for: DAPCy: a Python package for the discriminant analysis of principal components method for population genetic analyses
Source: Bioinform Adv. 2025 Jun 18;5(1):vbaf143. doi: 10.1093/bioadv/vbaf143 (PMC12237503; doi:10.1093/bioadv/vbaf143)
Supplement: vbaf143_Supplementary_Data [file vbaf143_supplementary_data.pdf]

Supplementary Information

1. DESCRIPTION OF THE METHODS

A. The Truncated Singular Value Decomposition (SVD)

Truncated SVD is a form of matrix factorization that approximates a given matrix A by retaining only the top k singular values and their corresponding singular vectors (Falini, 2022). Given a real or complex matrix A of size $m \times n$, the SVD of A is described as:

$$A = U\Sigma V^T \tag{S1}$$

where U is an $m \times n$ orthogonal matrix (left singular vectors), Σ is a $m \times n$ diagonal matrix (in descending order) on the diagonal, and V^T is a $n \times n$ orthogonal matrix (right singular vectors). In a truncated SVD, instead of keeping all singular values and vectors, we select only the top k singular values. The truncated SVD for rank k is formulated as:

$$A_k = U_k \Sigma_k V_k^T \tag{S2}$$

where U_k is an $m \times k$ orthogonal matrix consisting of the first k columns U, Σ_k is a $k \times k$ diagonal matrix with the top k singular values, and V_k^T is a $k \times n$ matrix consisting of the first k rows of V^T.

In the `scikit-learn` Python package, by default, the truncated SVD is estimated using the *randomized SVD* algorithm developed by Halko et al. (2010) which is efficient for large-scale matrices where computing the full SVD is impractical. Given a matrix $A \in \mathbb{R}^{m \times n}$, a target rank k, and a small oversampling parameter p, the randomized SVD algorithm computes an approximate rank-k decomposition of A as follows:

1. **Random projection:** Draw a random matrix

   $$\Omega \in \mathbb{R}^{n \times (k+p)},$$

   whose entries are typically i.i.d. Gaussian or uniformly distributed. Form the matrix

   $$Y = A\,\Omega \ \in \ \mathbb{R}^{m \times (k+p)}.$$

   This "sketch" Y captures the dominant action of A in a lower-dimensional subspace.

2. **Compute an orthonormal basis:** Factor Y via a QR decomposition:

   $$Y = Q\,R,$$

   where $Q \in \mathbb{R}^{m \times (k+p)}$ has orthonormal columns, and $R \in \mathbb{R}^{(k+p) \times (k+p)}$ is upper triangular. The columns of Q form an approximate basis for the column space of A.

3. **Form the reduced matrix:** Project A onto this basis:

   $$B = Q^T A, \quad B \in \mathbb{R}^{(k+p) \times n}.$$

   By construction, B is much smaller than A when $k \ll m, n$.

4. **Compute the SVD of the smaller matrix:** Perform a standard SVD on B:

   $$B \ = \ \widetilde{U}\,\Sigma\,V^T, \quad \widetilde{U} \in \mathbb{R}^{(k+p) \times (k+p)}, \quad \Sigma \in \mathbb{R}^{(k+p) \times n}, \quad V \in \mathbb{R}^{n \times n}.$$

5. **Lift back to dimension m:** Define

   $$U \ = \ Q\,\widetilde{U} \quad \in \mathbb{R}^{m \times (k+p)}.$$

   An approximate factorization of A is then

   $$A \ \approx \ U\,\Sigma\,V^T.$$

6. **Truncate to rank k:** Keep only the top k singular values (and corresponding columns in U and V):

$$U_k = U(:, 1{:}k), \quad \Sigma_k = \Sigma(1{:}k, 1{:}k), \quad V_k = V(:, 1{:}k).$$

   Thus the *rank-k approximation* to A is given by

$$A_k = U_k \Sigma_k V_k^T,$$

   which captures the largest k singular values and corresponding singular vectors.

B. The Sum of Squared Errors (SSE) in K-means Clustering

The SSE estimates the total squared distance between each data point and the centroid of its assigned cluster. For a set of N data points $\{x_1, x_2, \ldots, x_N\}$ and K clusters with centroids $\{\mu_1, \mu_2, \ldots, \mu_K\}$, the SSE is given by:

$$SSE = \sum_{i=1}^{K} \sum_{x \in C_i} \|x - \mu_i\|^2 \tag{S3}$$

where C_i is the set of data points belonging to the i-th cluster, μ_i is the centroid (mean) of cluster C_i, and $\|x - \mu_i\|^2$ the squared Euclidean distance between the point x and the centroid μ_i.

C. The Elbow Method

The elbow method is a heuristic used to determine the optimal number of clusters by plotting the SSE values for different numbers of clusters and identifying a point where the rate of decrease sharply slows down, resembling an "elbow" (Ikotun et al. 2023; Thia 2022). This point typically indicates a balance between the number of clusters and the variance explained:

- **Plot SSE vs. Number of Clusters:** calculate SSE for a range of cluster numbers and plot the results.
- **Identify the Elbow Point:** look for a point on the plot where the SSE begins to decrease more slowly, forming an elbow shape. This point suggests the optimal number of clusters.

D. Silhouette Scores

The Silhouette score is a measure of how similar an object is to its own cluster (cohesion) compared to other clusters (separation) (Shutaywi et al., 2021). For a data point i, the Silhouette score $s(i)$ is calculated as:

$$s(i) = \frac{b(i) - a(i)}{max(a(i), b(i))} \tag{S4}$$

where $a(i)$ is the average distance between i and all other points in its own cluster (intra-cluster distance), and $b(i)$ is the average distance between i and the points in the nearest neighboring cluster (inter-cluster distance).

The silhouette score ranges from -1 to 1:

- A value close to 1 indicates that the data point is well clustered.
- A value close to 0 indicates that the data point is on or very close to the decision boundary between two neighboring clusters.
- A value close to -1 indicates that the data point might have been assigned to the wrong cluster.

The average Silhouette coefficient over all data points provides an overall measure of clustering quality. Higher average Silhouette values indicate better-defined clusters.

2. BENCHMARKING AND POPULATION STRUCTURE ANALYSES

A. Description of the Datasets and Quality Control

To assess the model performance of DAPCy, we used as test datasets the *Plasmodium falciparum* v7 (Pf7; MalariaGEN, 2023) and the 1000 Genomes Project phase 3 release datasets (1KG; The 1000 Genomes Project Consortium, 2015). From Pf7, we used 16,203 sample quality-controlled “PASS” samples as indicated in the sequencing metadata. The 1KG dataset includes genomic data with 2,505 samples across 26 populations, representing five genetic population ancestries: European, Admixed, African, East Asian and South Asian.

For both datasets, we used `PLINK`[1] to filter SNPs with minor allele frequency (MAF < 0.10) and linkage disequilibrium (LD, $r^2 < 0.3$) using the options `'--maf 0.1'` and `'--indep 50 1 1.5'`. This filtering was performed to reduce sample size and retain uncorrelated variants.

B. Benchmarking

We assessed the runtime and memory usage of DAPCy as a function of sample and feature size (number of SNPs), using up to 120 PCs from the DAPC method. The dataset was split into training and test sets, with 70% of the data used for training and 30% reserved for testing to evaluate the tool’s performance. In addition, we compared `DAPCy`, including k_{CV}-fold cross-validation, with the R implementation of the DAPC method from the package `adegenet`, using the function `xvalDapc()` with bootstrapping cross-validation. For classification, we used the country of origin as population groups. We assessed performance using a range of up to 120 PCs with 10 replicates, specified using the option `n.rep = 10`. Finally, to evaluate model performance, we estimated the mean accuracy across different training splits (from 50% to 90% of the full dataset).

Analyses were run on a system with AMD 96 Cores (500 Gb RAM), 64-bit Ubuntu Linux 20.04.6 LTS. In all analysis, we only focused on measuring the wall time for the DAPC method.

C. Population structure using the DAPC method based on data labels

For population structure analysis on the Pf7 and 1KG datasets, we followed the guidelines provided by Thia (2022) for the inference of genetic groups using the DAPC method. First, we performed a classification analysis assuming a prior expectation of the number of populations (k_{prior}). For the Pf7 dataset, we used the country of origin as groups labels ($k_{\text{prior}} = 33$) whereas for the 1KG dataset we used the genetic population groups ($k_{\text{prior}} = 5$). As input for the DAPC method, we selected the number of principal components as k_{prior}. For cross-validation and grid search, we used k_{CV}-fold cross validation with $k_{\text{CV}} = 10$ to select the optimal number of PCs.

D. *De novo* inference of population groups

For the Pf7 dataset, we applied the K-means clustering algorithm for the inference of population groups, following the guidelines from Thia (2022), assuming that the effective number of populations is unknown (k_{infer}). As input parameters, we used 33 PCs with clusters ranging between 2 and 100. Using the Elbow method and Silhouette scores, we then selected the optimal number of clusters as k_{infer} and assessed their quality. To corroborate the obtained results, we also obtained k_{infer} by examining the PC screeplots from the PCA analysis. As described by Thia (2022), we infer the number of population groups by selecting the PC that creates an "elbow-shaped" pattern from the explained variances. As input for the DAPC method, we selected the number of principal components following the $\text{PC}_{opt} = k - 1$ criterion as predictors (Patterson et al., 2006; Thia, 2022).

3. SUPPLEMENTARY RESULTS

A. Memory and Runtime Performance

[1] https://www.cog-genomics.org/plink

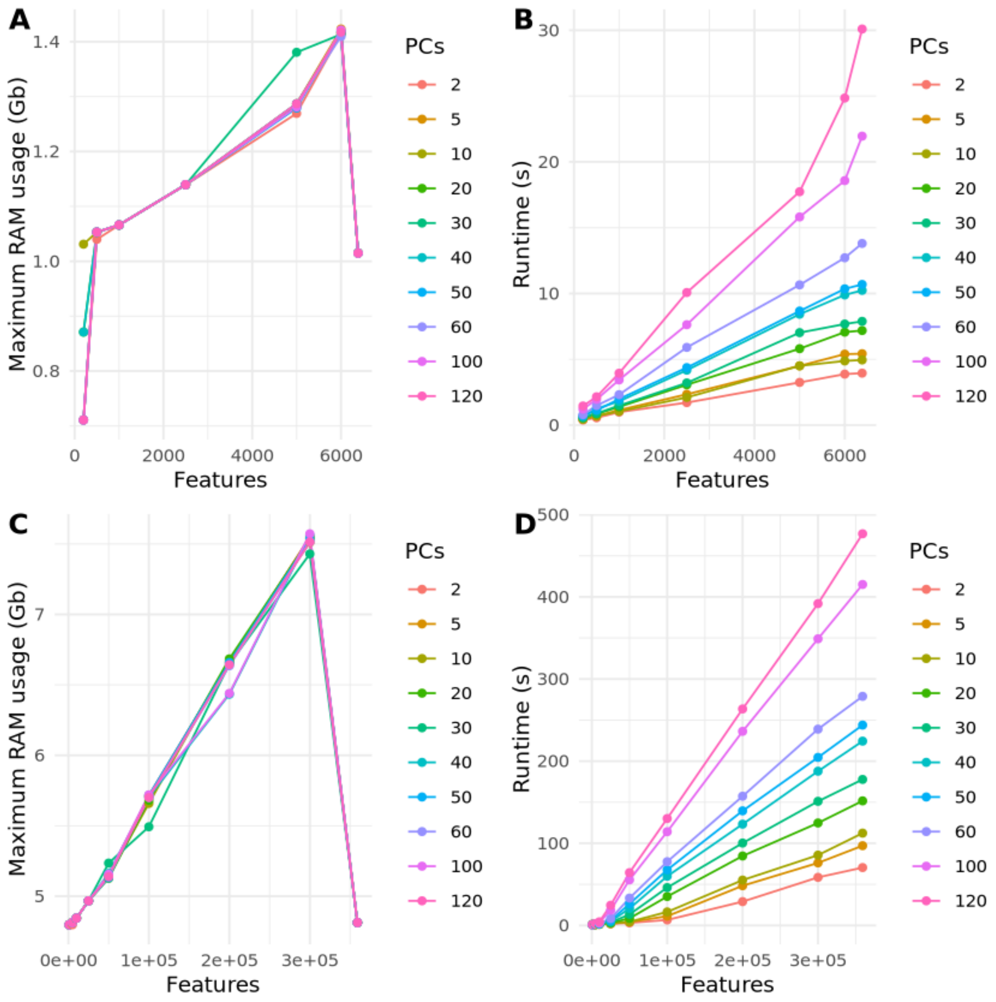

Fig. S1. Performance of DAPCy on the Pf7 ($N = 16,203$) and 1KG ($N = 2,505$) datasets as a function of feature size (SNPs) at different PCs. A Memory usage (GB) for Pf7 dataset. B Runtime (s) for Pf7 dataset. C Memory usage (GB) for 1KG dataset. D Runtime (s) for 1KG dataset.

B. Population Structure Analyses

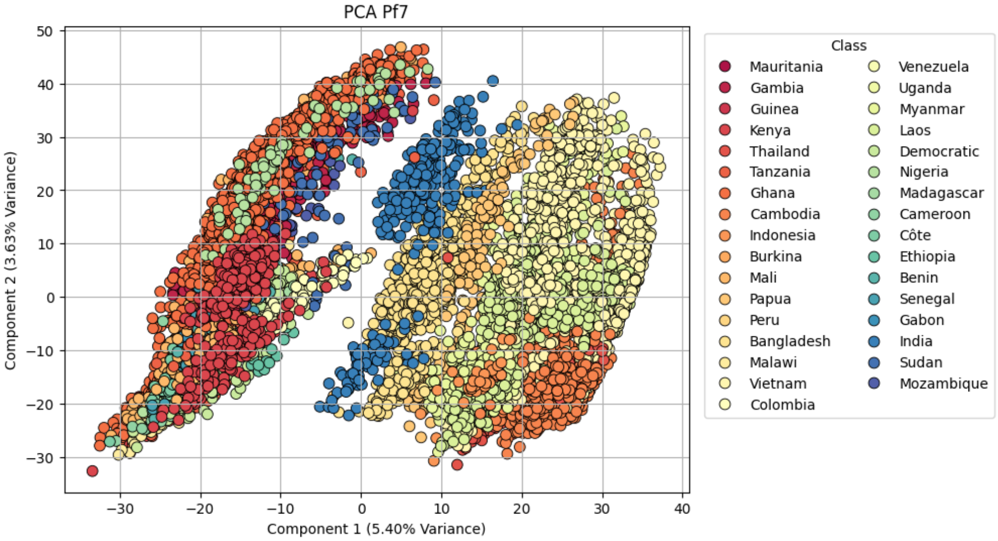

Fig. S2. First two principal components estimated from the Pf7 dataset using country of origin as labels ($k_{\text{prior}} = 33$)

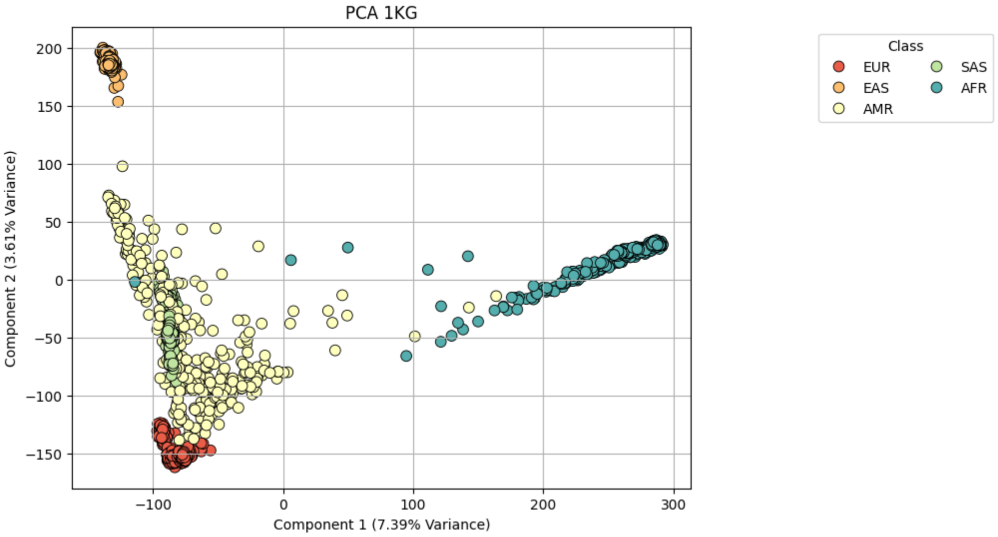

Fig. S3. First two principal components estimated from the 1KG dataset using genetic population groups as labels ($k_{\text{prior}} = 5$).

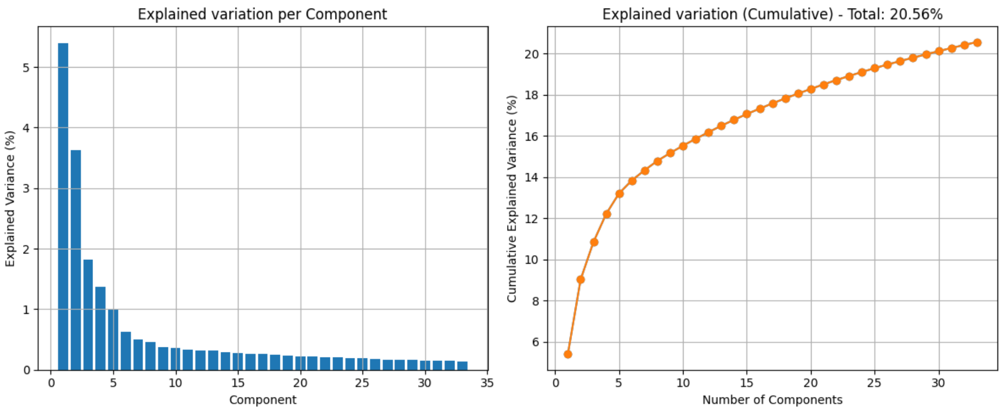

Fig. S4. Cumulative variance from the Principal Component Analysis on the Pf7 dataset. From the explained variances per component, the inflexion point is observed at the fourth PC.

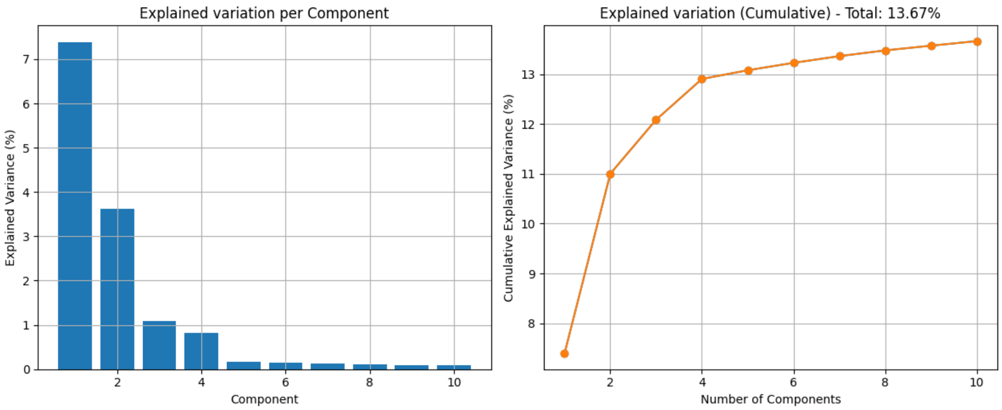

Fig. S5. Cumulative variance from the Principal Component Analysis on the 1KG dataset. From the explained variances per component, the inflexion point is observed at the fourth PC.

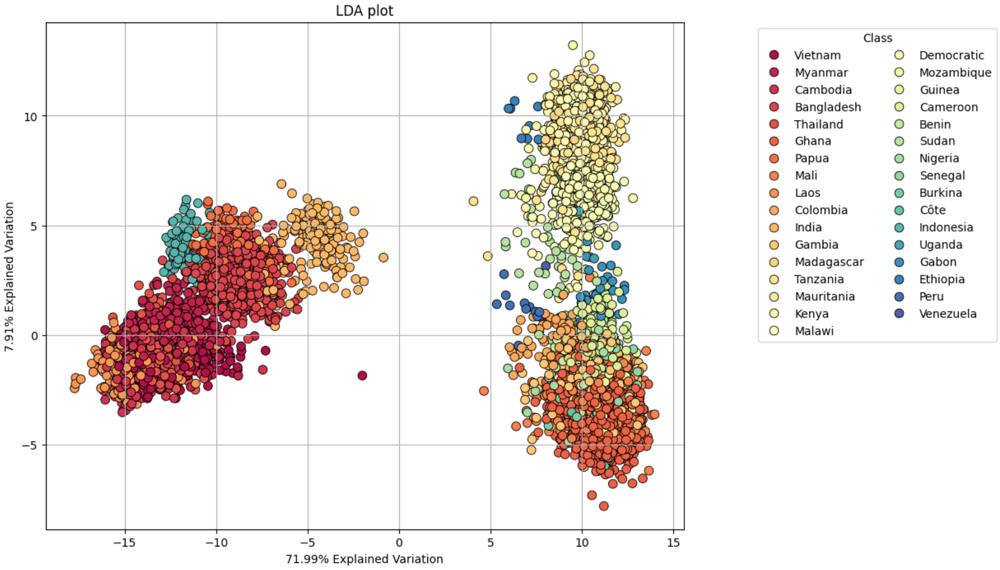

Fig. S6. First two discriminant components estimated from the Pf7 training dataset using country of origin as labels (Linear Discriminant Analysis). DAPCy was applied with PCs = 32 obtained with grid search k_{CV}-fold cross validation ($k_{\mathrm{CV}} = 10$). Accuracy score = 71.86%.

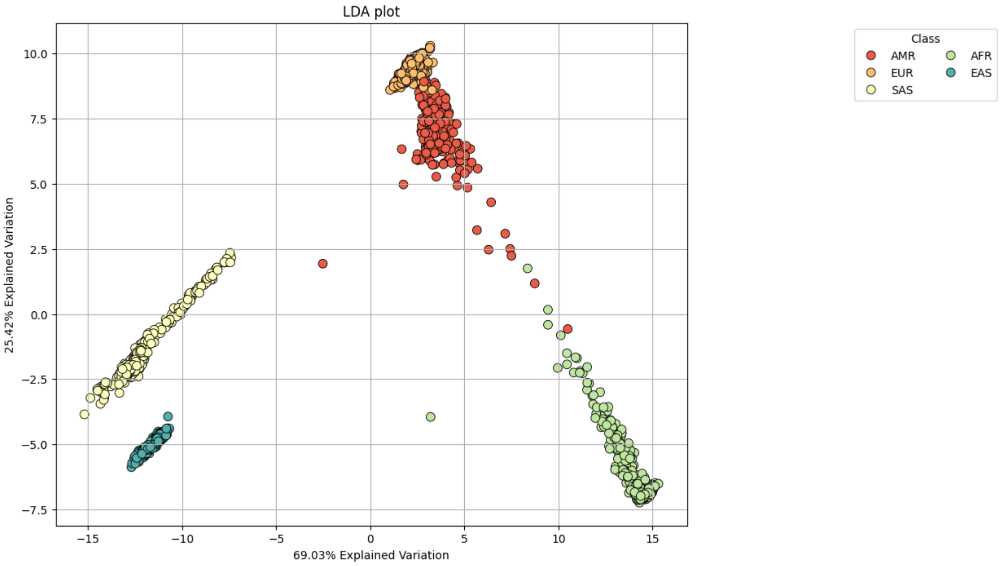

Fig. S7. First two discriminant components estimated from the 1KG training dataset using country of origin as labels (Linear Discriminant Analysis). DAPCy was applied with PCs = 3 obtained with grid search k_{CV}-fold cross validation ($k_{\mathrm{CV}} = 10$). Accuracy score = 97.50%.

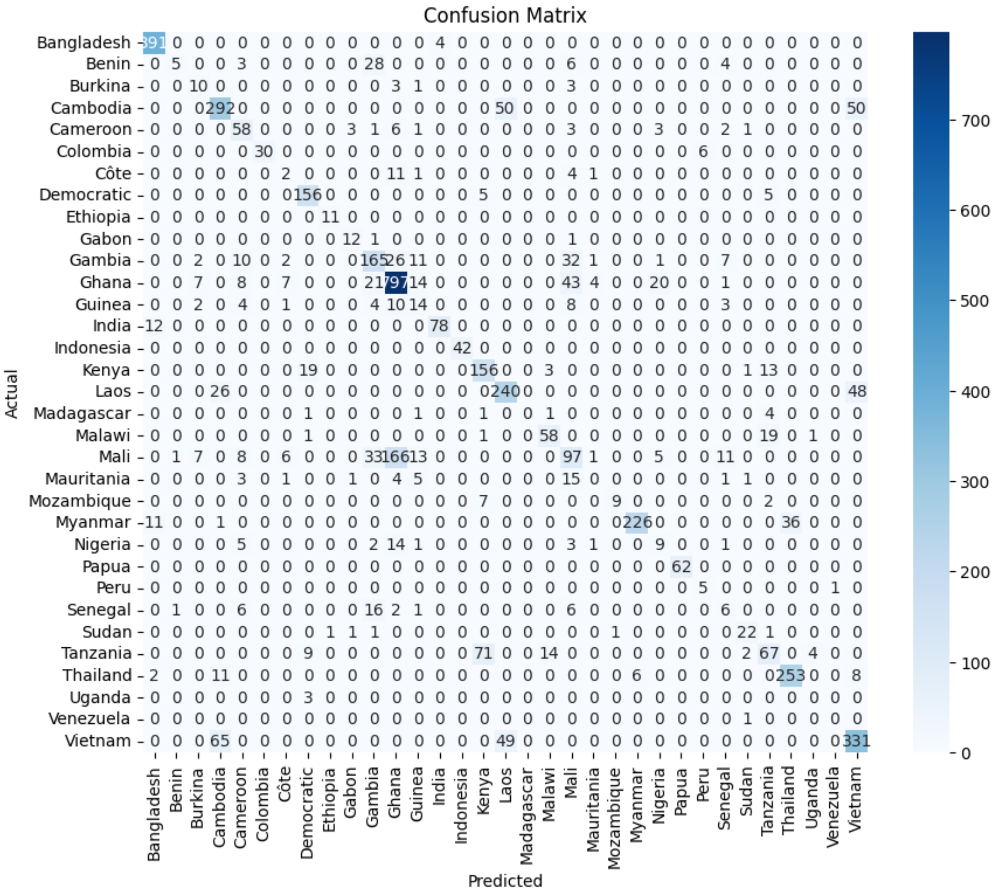

Fig. S8. Confusion matrix generated for the Pf7 using the test dataset.

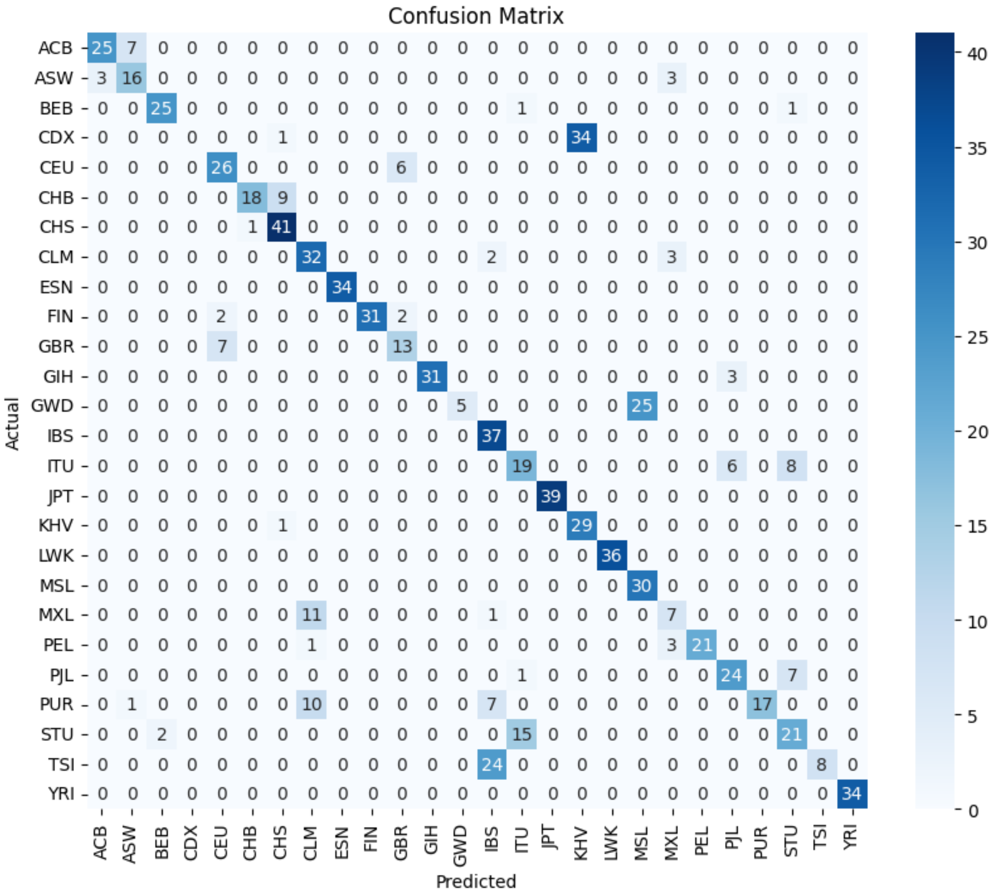

Fig. S9. Confusion matrix generated for the 1KG using the test dataset.

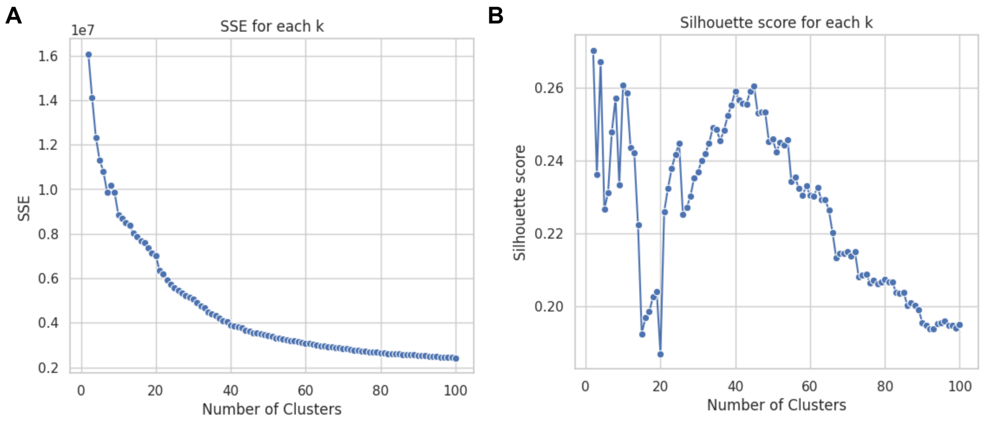

Fig. S10. Model diagnostics of the K-means clustering applied to the Pf7 dataset (k = *100*). **(A)** Elbow method. **(B)** Silhouette scores. For Silhouette scores, the strongest separation is seen at $k_{\text{infer}} = 2$. However, the second-highest Silhouette score occurs at $k_{\text{infer}} = 4$, which aligns with the inflection point identified by the Elbow method.

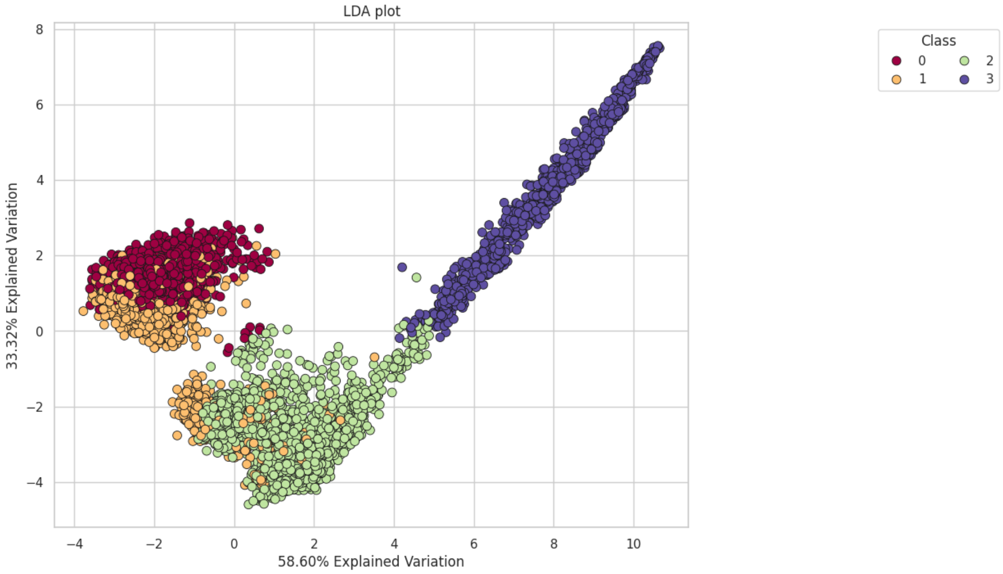

Fig. S11. First two discriminant components estimated from the Pf7 training dataset using $k_{\text{infer}} = 4$ (Linear Discriminant Analysis). DAPCy was applied with PCs = 3, based on the k - 1 criterion, with k_{CV}-fold cross validation ($k_{\text{CV}} = 10$). Accuracy score = 95.76%.

REFERENCES

1. Antonella Falini. A review on the selection criteria for the truncated SVD in Data Science applications. *Journal of Computational Mathematics and Data Science*, 5:100064, 2022.
2. Abiodun M. Ikotun, Absalom E. Ezugwu, Laith Abualigah, Belal Abuhaija, Jia Heming K-means clustering algorithms: A comprehensive review, variants analysis, and advances in the era of big data. *Information Sciences*, 622:178-210, 2023.
3. Meshal Shutaywi, Nezamoddin N. Kachouie Silhouette Analysis for Performance Evaluation in Machine Learning with Applications to Clustering. *Entropy*, 23:759, 2021.
4. Joshua A. Thia. Guidelines for standardizing the application of discriminant analysis of principal components to genotype data. *Molecular Ecology Resources*, 23:5239-38, 2022.

5. MalariaGEN. Pf7: an open dataset of Plasmodium falciparum genome variation in 20,000 worldwide samples. *Wellcome Open Research*, 8, 2023.
6. The 1000 Genomes Project Consortium A global reference for human genetic variation *Nature*, 26:68-74, 2015.
7. The 1000 Genomes Project Consortium An integrated map of genetic variation from 1,092 human genomes *Nature*, 491:56-65, 2012.
8. Nathan Halko, Per-Gunnar Martinsson, Joel A. Tropp Finding structure with randomness: Probabilistic algorithms for constructing approximate matrix decompositions *arXiv*, arXiv:0909.4061, 2010.
